# The Performance and Synthesis of Alkynyl−Functionalized Benzoxazine Containing Phthalide Side Groups and Cyano Groups with Different Molecular Weights

**DOI:** 10.3390/polym15163478

**Published:** 2023-08-20

**Authors:** Nianjun Kang, Shuai Yang, Xuhai Xiong, Anchang Han, Rong Ren, Jing Wang

**Affiliations:** 1 Liaoning Key Laboratory of Advanced Polymer Matrix Composites Manufacturing Technology, Shenyang Aerospace University, Shenyang 110136, China; kangnianjun1@crcchem.com (N.K.); yangshuai2@stu.sau.edu.cn (S.Y.); hananchang0516@163.com (A.H.); renrongsy@126.com (R.R.); jingwang_1217@126.com (J.W.); 2 School of Materials Science and Engineering, Shenyang Aerospace University, Shenyang 110136, China

**Keywords:** benzoxazine, alkynyl, curing mechanism, cyano group

## Abstract

Benzoxazine resins are widely employed in a variety of applications due to their exceptional heat resistance and treatment properties. However, traditional benzoxazine resins still confront hurdles in today’s engineering applications, such as their inability to provide long-term service in high-temperature settings and their inadequate toughness. In this study, four alkyne-functionalized benzoxazines with phthalide side groups and cyano groups of varying molecular weights were produced. Fourier transform infrared spectroscopy (FT-IR) and hydrogen nuclear magnetic resonance spectroscopy (^1^H-NMR) were used to characterize the resin structure, and differential scanning calorimetry (DSC) was used to investigate the thermal curing kinetics at different warming rates. The apparent activation energy was 116.9 kJ/mol. In-situ FT-IR was used to investigate the cure mechanism. Dynamic mechanical analysis (DMA) was used to evaluate the gelation time of BOZ series resins at various temperatures, and the curing process was designed by combining the results with DSC. The Tg of the composites made using BOZ-1N21 as the matrix was 336 °C, which was much higher than the Tg of the BP-a resin made with aniline, phenolphthalein, and formaldehyde (Tg = 251 °C). As a result, the resin system is expected to be employed in applications requiring high-temperature resistance and toughness.

## 1. Introduction

Benzoxazine resins are a relatively new class of thermoset phenolic resins developed in the last decade that combine the superior high temperature resistance and acid and alkali resistance of phenolic resins with the remarkable mechanical properties and manufacturability of epoxy resins [1,2,3]. They are characterized by the mechanism of ring cleavage and curing crosslinking during the curing process, which does not produce small molecules and results in low curing shrinkage, enabling the production of products with improved heat resistance and mechanical properties compared to conventional phenolic resins [4]. Due to its various advantageous properties, it holds enormous potential for applications under high-temperature conditions, such as high-temperature-resistant shells for rocket engines and flame-retardant materials for aircraft, as well as other fields.

Benzoxazine resins were first synthesized by Cope and Holly [5] in 1944 through condensation reactions between primary amines and formaldehyde with phenol, but the potential of polybenzoxazine was not fully realized until much later. Riess et al. [6] demonstrated the regioselectivity of the preferential reaction of the oxazine ring with phenolic compounds. Ishida and colleagues further advanced the study of monomeric benzoxazine by inventing a solvent-free synthesis. These studies showed that various structures of benzoxazine monomers can be achieved by precisely designing the benzoxazine monomers with different functionalities in combination with various phenols and amines. This provides a theoretically sound basis for the modification of benzoxazine resins to obtain polymeric materials with desired properties.

The main obstacle to the development and application of benzoxazine resins is a common problem with other high-temperature resins, namely the tendency to become easily brittle and to exhibit low toughness in the cured products. The primary approach to overcoming this problem is to develop a novel molecular structure, which includes increasing the molecular weight of the monomer, thus reducing the density of curing crosslinks, and improving the toughness of the resins without affecting high temperature resistance to the greatest extent possible. In order to achieve this, novel polymeric-based precursors have been synthesized by incorporating benzoxazine units either as side chains, end chains, or in the main chain of the polymer. A new crosslinkable telechelic containing a benzoxazine moiety at the chain end with good ductility was synthesized by Masanori et al. [7]. In the last two decades, research on benzoxazine resins has focused particularly on (i) chemical modification/molecular design, such as the incorporation of phenylacetylene groups, cyano groups [8,9], allyl [10], propargylether [11], etc.; (ii) modification by blending with other resins (including epoxy resins, bimatic resins [12], etc.); and (iii) cure kinetics and mechanistic analysis [13]. It was found that the addition of alkyne groups can increase the number of functional groups in the benzoxazine molecule that can be crosslinked [14]. In addition, the alkyne groups formed a six-membered ring structure during curing and crosslinking, increasing the crosslinking density of the resin and thus its Tg, which gave the resin higher heat resistance [15,16]. This excellent thermal property is of great advantage in terms of heat resistance and ablation [17]. The characteristics that ensure excellent thermal and mechanical properties of the resin can be provided by the rigid and stable triazine rings formed by cyano groups. In their review, Yagci et al. [18] mentioned that the aminomethyl (−CH2−NR−CH2−) group in polybenzoxazine is the weakest point of the network structure. In order to improve thermal stability, nitrile-functionalized monomers are an important modification method. In the studies by Zhang S. et al., the Tg of high-performance resins prepared from benzoxazine monomers, cyano groups, and alkyne groups is 292.7 °C [19,20,21,22,23]. The presence of phenolphthalein structures can effectively reduce the proportion of crystal regions in the resin structure, thereby increasing toughness. At the same time, the cyclic structure of phenolphthalein also makes an important contribution to the heat resistance of the resin. R. M. Ghetiya et al. [24] synthesized two benzoxazines containing bisphenol C (BCO) and phenolphthalein (PHO) using bisphenol C/phenolphthalein, formaldehyde, and aniline. The residual percentage of the phenolphthalein samples (56–67%) was significantly higher than that of the bisphenol C samples (20–25%) at 550 °C. Above all, the above work is still difficult to meet the usage needs in the high-end field. Therefore, according to the concept of molecular structure design and based on the previous three, it is of great significance to prepare alkynyl-functionalized benzoxazine containing phthalide side groups and cyano groups.

In this study, the curing behaviors of BOZ-1N were investigated by DSC and FT-IR, which suggested that the cyclization reaction of the alkynyl group and the ring cleavage reaction of the benzoxazine ring occur simultaneously, and the conversion rate of the alkynyl group is significantly lower than that of the benzoxazine ring during the curing process. The thermal properties and structures of the BOZ-1N system were investigated systematically. The results showed that the cured resins possessed high glass transition temperatures and excellent thermal properties. Meanwhile, the cyano group absorption of the polymer network was observed in IR spectra, indicating the cyano group was almost unconsumed in the curing process. Interestingly, the storage modulus showed an upward trend around 325 °C in DMA testing. This may be because triazine has been formed in the polymer network, and the curing mechanism follows the traditional curing system with Lewis acids/bases as agents [25]. The molecular weight of monomers also has a certain impact on their heat resistance performance, so molecular weight will be discussed as a variable in relation to performance.

## 2. Materials and Methods

### 2.1. Raw Materials

Phenolphthalein (PP), 3-ethynylaniline, 2,6-Dichlorobenzonitrile, formaldehyde, acetone, anhydrous potassium carbonate, deionized water, and anhydrous ethanol were purchased from Shenyang Xinhua Reagent Factory, while diphenylmethane bismaleimide and o, o’-diallyl bisphenol A were purchased from Honghu Shuangma New Material Technology Co. (Honghu, China). All reagents were of analytically pure quality and were used as received. Finally, T700 carbon fiber was obtained from Toray Corporation, Tokyo, Japan.

### 2.2. Synthesis of BOZ-1N Series Resin

**Figure 1:**
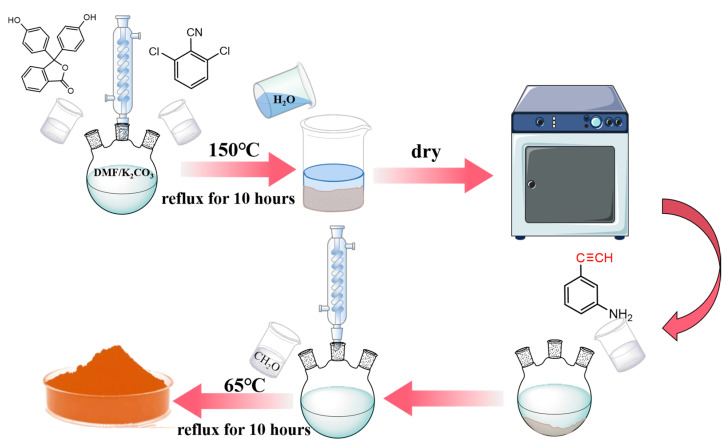
Synthesis flow diagram.

According to the dosage given in Table 1, the required amounts of phenolphthalein, 2,6-Dichlorobenzonitrile, and anhydrous potassium carbonate were weighed and then added to a three-neck flask containing DMF as a solvent. Then the stirrer was activated, and the reaction solution was heated to 150 °C and kept at reflux for 10 h. The reaction solution was filtered off while it was still hot, and deionized water was added to the filtrate until the precipitate began to stratify. The filter residue was then washed and dried to obtain phenol-hydroxyl-sealing polyarylether-cyano oligomers. The flow diagram is shown in Figure 1

The phenol-hydroxyl-sealing polyarylether-cyano oligomers synthesized in the previous step were dissolved in acetone containing 3-ethynylaniline (molar ratio 1:2), then formaldehyde was added, and the reaction was carried out at 65 °C under reflux for 10 h. At the end of the reaction, the reaction solution was poured into a rotary evaporator to evaporate and dry. Four BOZ-1N resins with different molecular weights (BOZ-1N21, BOZ-1N32, BOZ-1N43, and BOZ-1N98) were obtained, which appeared as brown powder solids.

### 2.3. Preparation of T700/BOZ−1N Composite Laminate

The BOZ-1N resin was dissolved in ethanol and configured into a resin solution with a mass fraction of 30%. This solution was then used for the impregnation process to produce a T700/BOZ-1N prepreg, followed by a molding process to produce a unidirectional composite plate. The curing process of the CF/BOZ-1N composite is 180 °C/2 h, 208 °C/3 h, 240 °C/3 h, and 260 °C/2 h.

### 2.4. Representation

DSC profiles were recorded using a PerkinElmer Diamond DSC instrument at various heating rates under a nitrogen atmosphere.

FT-IR spectra were recorded using a PerkinElmer Spectrum 100 FT-IR spectrometer with an average signal of 4 scans and collected in the wavenumber range of 4000 to 450 cm^−1^.

Varian Unity INOVA 400 NMR was used to test the ^1^H-NMR and ^13^C-NMR of BOZ series resins using deuterium chloroform (CDCl_3_) as a solvent and tetramethylsilane (TMS) as an internal reference. The observed frequency is 400 MHz, and chemical shifts are expressed in ppm.

DMA was performed using a TA Q800 single cantilever beam model with a drive frequency of 1 Hz and a heating rate of 5 °C/min ranging from 30 to 400 °C.

The gelation time (t_gel_) was measured by isothermal DMA in the single cantilever model with T700/BOZ-1N prepregs wrapped in tin foil paper at temperatures of 208 °C and 240 °C, respectively.

## 3. Results

### 3.1. Chemical Structural Characterization of BOZ Series Resins

**Figure 2:**
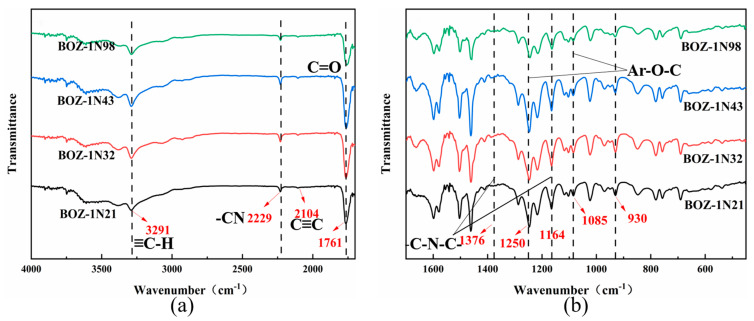
(**a**) FTIR spectra of BOZ-1N series benzoxazine resin; (**b**) Enlarged view of the FTIR spectra of BOZ-1N in the range 400 to 1700 cm^−1^.

FTIR appears to be an efficient method for quantitative as well as qualitative curing investigation and structural characterization of resins. The FTIR spectra of BOZ-1N resin can be seen in Figure 2. The −C≡C and ≡C−H stretching vibrations of the alkynyl groups are responsible for the observed weak peak at 2104 cm^−1^ and strong peak at 3291 cm^−1^ on the four curves, respectively. The vibrational absorption peak of lactone carbonyl (C=O) in the cardo ring structure is 1761 cm^−1^. Both 1376 cm^−1^ and 1164 cm^−1^ stretching vibrations of −C−N−C in the imide ring are recorded. The absorption at 1250 cm^−1^ and 1085 cm^−1^ might be attributed to the Ar−O−C stretching mode of the oxazine ring. Furthermore, the absorption peak at 930 cm^−1^ is widely utilized to identify oxazine ring formation [26,27]. The −CN groups’ characteristic absorption peaks occur at 2229 cm^−1^, demonstrating the effective production of BOZ-1N resin. Furthermore, no influence of the varied molar ratios of 2,6-Dichlorobenzonitrile and phenolphthalein on the structure of the resin products was found, and their FT-IR spectra were almost comparable.

**Figure 3:**
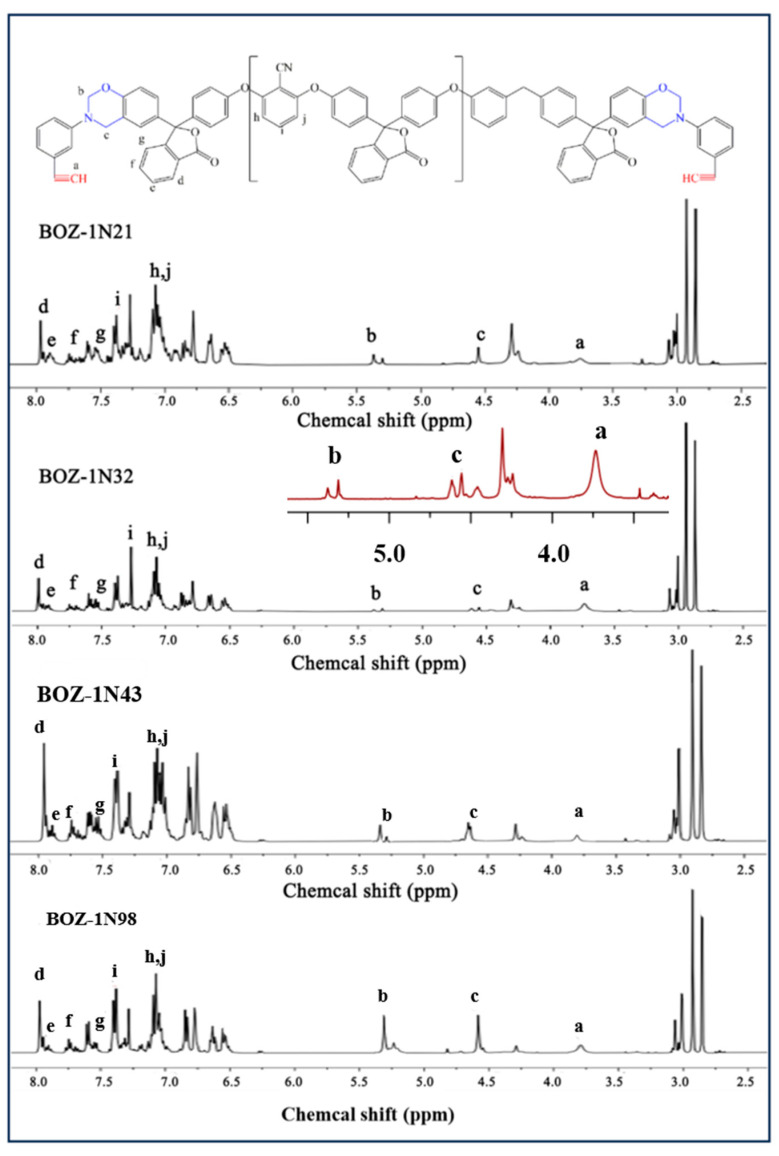
^1^H-NMR spectrum of BOZ-1N resin.

**Figure 4:**
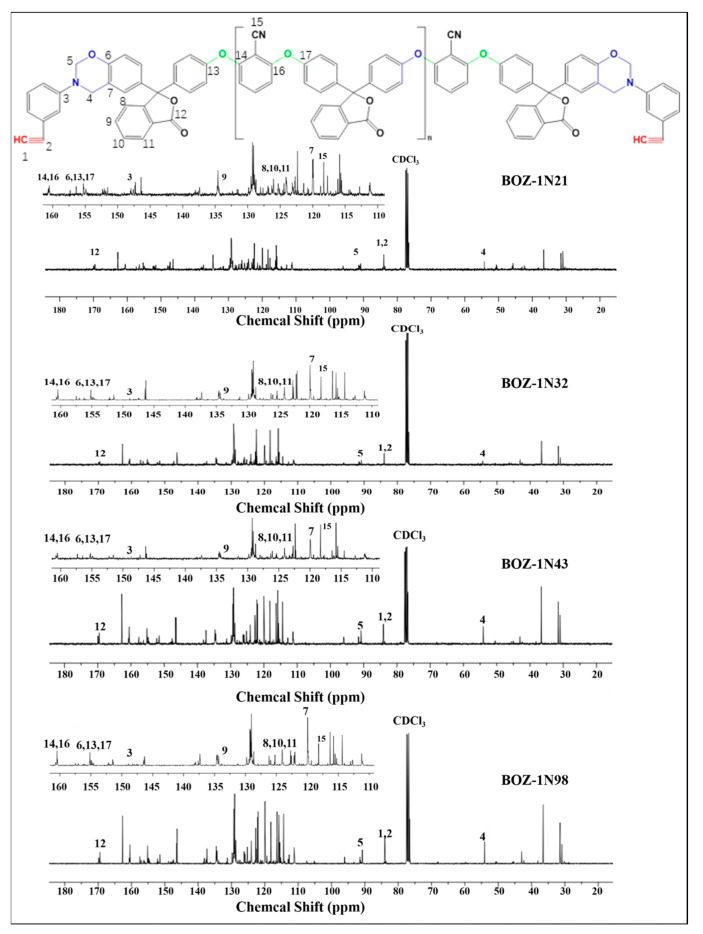
^13^C-NMR spectrum of BOZ-1N resin.

The ^1^H-NMR spectra of four distinct molecular weights of BOZ-1N are shown in Figure 3. Because of the introduction of the characteristic functional groups ether bond, acetylene groups, and cyano groups into the structure, as well as the different molecular weights, the content of each functional group in the resin molecule varies, resulting in more complicated ^1^H-NMR spectra of the four BOZ-1N, but the positions of each peak are essentially the same. Figure 3 reveals that the methylene of the oxazine ring has resonance peaks at 4.61 ppm and 5.39 ppm, respectively, while the proton of the acetylene groups [28,29] has a resonance peak at 3.74 ppm. The resonance peaks at 7.11 ppm and 7.42 ppm in benzonitrile groups are caused by protons on the benzene ring, while the resonance peaks at 7.64 ppm, 7.81 ppm, and 7.96 ppm demonstrate the presence of the phenolphthalein structure. It is worth noting that the intensity of the methylene proton peaks b and c on the oxazine ring of BOZ-1N32 is very low compared with other resins, but it does not mean that the benzoxazine ring is not formed, and the peaks can still be clearly seen in the enlarged image, and the intensity of peaks b and c are comparable. Furthermore, the structure of BOZ-1N was confirmed by using ^13^C-NMR, and the corresponding chemical shifts are shown in Figure 4. The 83.27 ppm resonance peak is attributed to the carbon atom in the acetylene group; 155.07 ppm and 161.04 ppm are the peaks of the carbon atoms in the two C−O−C structures of the Ar−O−Ar moiety, respectively; the 117.09 ppm resonance peak originates from the carbon atom in the cyano group; the 90.07 ppm is attributed to the carbon atoms in the O−C−N in the oxazine ring; and the Ar−C−N resonance peak in the oxazine ring is located at 54.24 ppm.

By combining the FT-IR, ^1^H-NMR, and ^13^C-NMR spectra, it is possible to conclude that the target product was successfully made and that the molar ratio of Phenolphthalein and 2,6-Dichlorobenzonitrile merely alters the size of the molecular weight with no discernible influence on the product structure.

### 3.2. Intrinsic Viscosity and Average Molecular Weight of BOZ-1N Benzoxazine Resin

**Figure 5:**
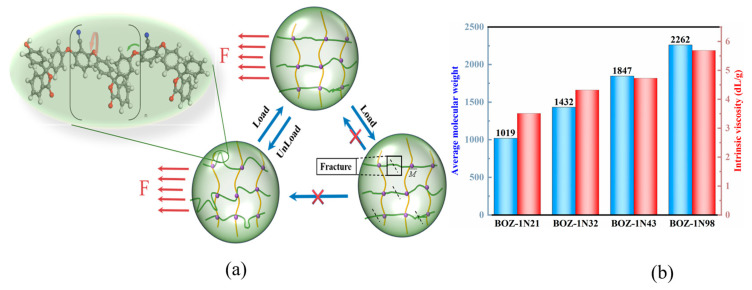
(**a**) The formation and destruction of resin toughness; (**b**) Intrinsic viscosity and average molecular weight of benzoxazine.

The toughness of the resin is connected to the crosslinking density and the length of the molecular chain, and the length of the molecular chain will affect the impact strength of the resin to some extent, as seen in Figure 5a. When the molecular chain with curled conformation in the monomer is affected by an external force, the force will not directly act on the weakest point in the molecular structure but will force the molecular chain to stretch and consume part of the force. When the load is unloaded, the molecular chain tends to curl to achieve the purpose of toughening due to the entropy increase principle.

The resins were evaluated for viscosity using an Ubbelohde viscometer in this study. Figure 5b depicts the intrinsic viscosity data for four distinct molecular weights. As expected, as the concentration of 2,6-Dichlorobenzonitrile increased, the intrinsic viscosity and average molecular weight of BOZ-1N resins increased significantly because 2,6-Dichlorobenzonitrile expanded the structure of the molecular chain in the system, causing greater friction between the molecular chains and increasing viscosity. Because of the curled molecular conformation and asymmetric structure, the crystal area of the molecular chain is reduced, and the entropy value is increased. When subjected to external forces, the energy can be converted into heat generated by the relative motion of the molecular chain, thereby increasing toughness.

### 3.3. Curing Behavior and Gelation Point Analysis of BOZ-1N21 Resins

**Figure 6:**
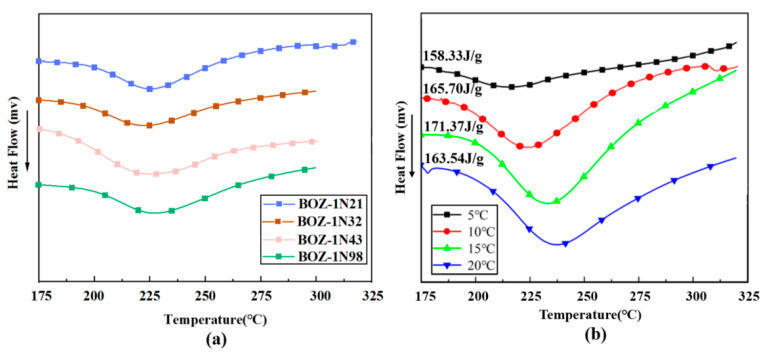
(**a**) DSC curves of BONZ-1N resins at 10 °C/min; (**b**) DSC curves of BOZ-1N21 at different heating rates.

DSC is a broad approach for investigating curing kinetics [30]. Non-isothermal approaches can be used to investigate the unknown cure mechanism. Figure 6a shows the DSC curves of four different BOZ-1N resins at a heating rate of 10 °C/min. It can be found that the characteristics of the four curves are basically the same, and the curing behaviors are comparable. Therefore, the curing kinetics of BOZ-1N21 resin are described as an example to discuss the curing behaviors at different heating rates. Figure 6b depicts the exothermic curves of BOZ-1N21 resin at various heating rates of 5 °C/min, 10 °C/min, 15 °C/min, and 20 °C/min. Due to the thermal hysteresis effect, these curves exhibit substantially wider exothermic peaks as the heating rate increases into the high temperature region. The curing properties are listed in Table 2.

The apparent activation energy (*Ea*) is a measure of the smallest amount of energy necessary for a resin to undergo a curing reaction, and its magnitude is one of the most critical parameters in determining the pace of the curing process. The Kissinger and Ozawa methods [31,32,33] were applied to calculate the kinetic parameters and build a kinetic model, which may be derived by combining the non-isothermal DSC curves (Figure 5) with the Kissinger (Equation (1)) and Ozawa (Equation (2)) methods.

Kissinger method:

(1) $$E_{a}=R\frac{d\ln(\beta /T_{p}^{2})}{d(1/T_{p})}\;\;$$

Ozawa method:

(2) $$E_{a}=0.95R\frac{d\ln(\beta )}{d(1/T_{p})}$$

where *β* is the rate of temperature rise (°C/min); *Tp* is the peak temperature (°C); *R* is the molar gas constant (8.314 J/mol·K); and *Ea* is the apparent activation energy (J/mol).

**Figure 7:**
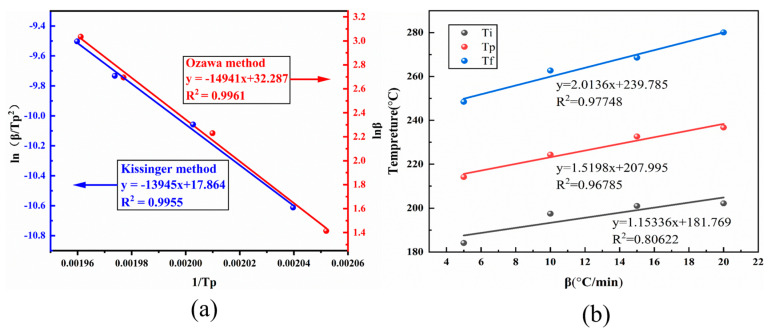
(**a**) Plots of *ln* (*β*/*T_p_*^2^) versus 1/*T_p_* and *lnβ* versus 1/*T_p_*; (**b**) Extrapolation of curing emperature when *β* = 0.

Scatter plots were constructed with *ln*(*β*/*T_p_*^2^) for 1/*Tp* and *ln*(*β*) for 1/Tp, respectively, based on the peak temperature Tp of DSC at varied heating rates of BOZ-1N21 resin, and the fitted curve computed by the Kissinger and Ozawa techniques similarly exhibited fairly linear connections. Figure 7a depicts the fitted pictures. The slopes of the pictures produced from the linear fits were entered into Equations (1) and (2) to calculate activation energies of 115.86 J/mol for the Kissinger equation and 117.94 J/mol for the Ozawa equation, with an average value of 116.90 J/mol for both equations. The gradient temperatures of 180 °C, 208 °C, and 240 °C for the curing cycle of the lower BOZ-1N21 resin at a temperature increase rate of 0 °C/min were determined using the linear extrapolation method (as shown in Figure 7b).

**Figure 8:**
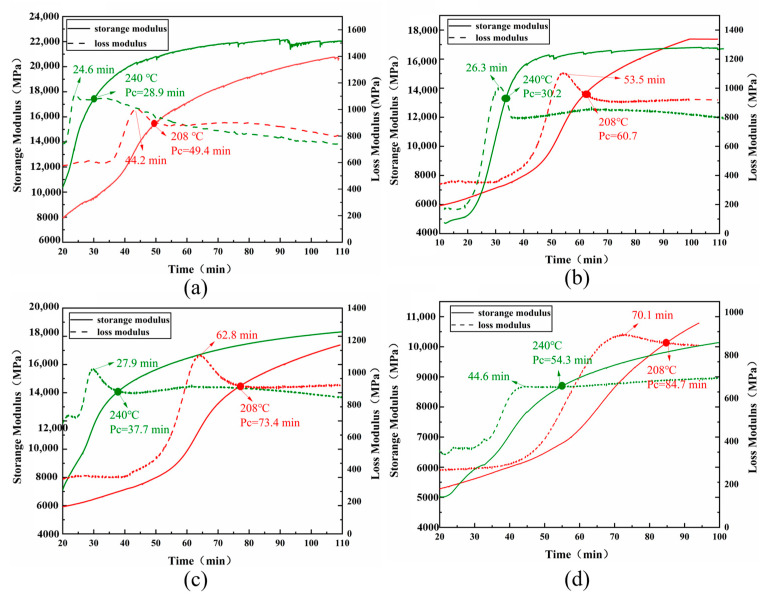
Gelation times of four BOZ-1N resins at 208 °C and 240 °C: (**a**) BOZ-1N21; (**b**) BOZ-1N32; (**c**) BOZ-1N43; (**d**) BOZ-1N98).

The most significant features of polymers are their thermodynamic properties [34]. Dynamic mechanical analysis (DMA) was used to evaluate the thermodynamic behavior of cured resins throughout various curing cycles. The gelation point (Pc) of a thermoset resin is an essential parameter that indicates the transition between the two phases during isothermal curing. DMA is a more accurate and adaptable instrument for measuring gelation time according to Tung’s theory and has been used in numerous resin systems [35,36,37]. When the curing system achieves the gelation state, the gelation point (Pc) is the junction of the storage modulus (G′) and loss modulus (G″) curves. Figure 8 depicts the temperature dependency of G′ and G″. The gelation of four T700/BOZ-1N prepregs coated in thin tin foil was measured using a single cantilever beam model at 208 °C and 240 °C, respectively.

Figure 8 illustrates that increasing the molecular weight of BOZ-1N resin delays the gelation point at the same curing temperature. The discrepancies in the cured network architectures induced by differing molecular weights are attributable to the aforementioned events. The cyano groups and phenolphthalein introduced into the molecular chain of BOZ-1N resins extend the length of the molecular chain; the higher the proportion of addition, the longer the molecular chain, which leads to a greater internal friction of the molecular chain when the gelation occurs, delaying the occurrence of the gelation; and the larger the proportion of the first two additions, the corresponding reduction in the points where crosslinking can occur, delaying the occurrence of the gelation. The storage modulus of the BOZ-1N98 resin system is lower than the first three, as shown in Figure 8d. This might be attributed to a drop in the proportion of alkyne groups, resulting in a decrease in crosslinking density and hence a change in storage modulus. As a result, the molecular chain length should be stretched to the greatest degree feasible, and the number of crosslinking points should be assured, resulting in a more solidified 3D molecular network system that enhances the stiffness of the composite material.

### 3.4. Curing Mechanism of BOZ-1N Resin

**Figure 9:**
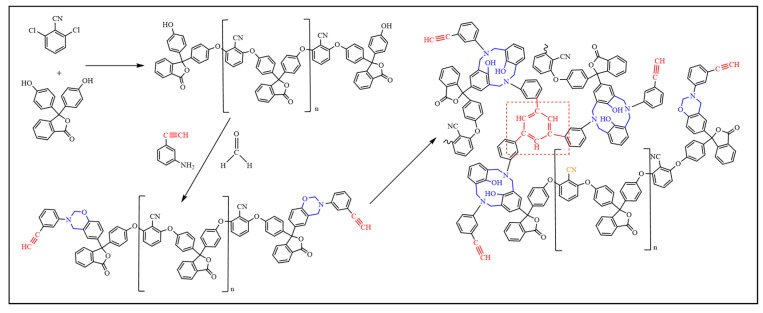
The main reactions during the curing process.

The biggest variation between the four resins is obviously their molecular weight; however, this has no effect on the curing procedure. Figure 9 shows the reactions involved in the curing process.

**Figure 10:**
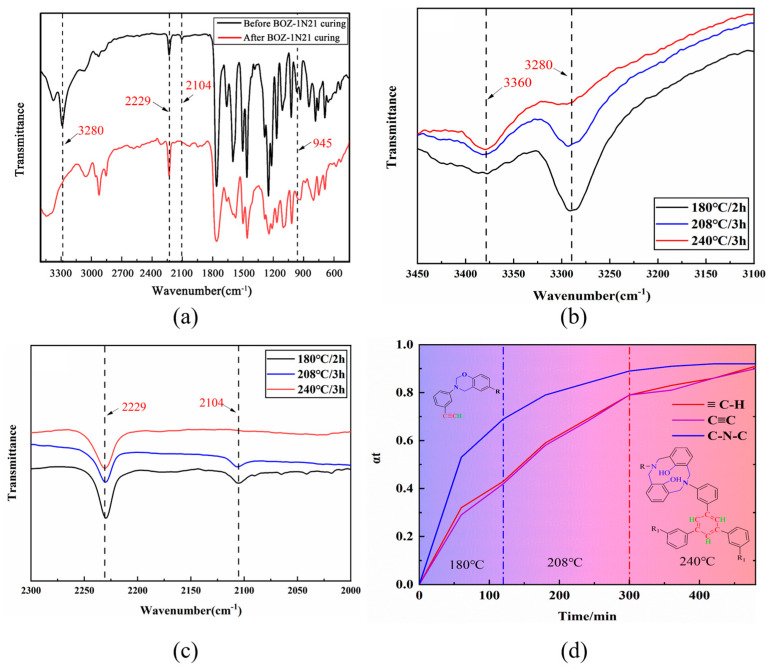
(**a**) FT-IR spectra of BOZ-1N21 resin before and after curing; (**b**,**c**) Isothermal FT-IR of BOZ-1N21 resin at different temperatures with curing time; (**d**) Conversion rate of functional groups at a specific temperature of BOZ-1N21 resin ($\alpha_{t}$).

In conjunction with Figure 6, all DSC curves of BOZ-1N21 resin at various heating rates contain just an exothermic peak. This suggests that the trigger conditions for oxazine ring cleavage polymerization and the alkynyl reaction coincide. In order to elucidate the complex curing mechanism of BOZ-1N resins, the in-situ isothermal infrared method was used to determine the curing process of resins, as shown in Figure 10.

Figure 10a depicts the changes in the characteristic peaks of BOZ-1N21 resin before and after curing. As the BOZ-1N21 resin cures, the alkyne groups eventually change into crosslinking or trimeric cyclization, causing the characteristic peaks at 3280 cm^−1^ and 2104 cm^−1^ to fade. Meanwhile, a new peak at about 3360 cm^−1^ is seen, which is caused by Ar-OH formed by oxazine ring cleavage. In Figure 10b,c, the strength of the characteristic peak signals of the aforementioned characteristic functional groups gradually increases or decreases with the degree of cure. These observations suggest that the terminal alkyne groups undergo simultaneous branching or trimerization cyclization events during oxazine ring cleavage polymerization. The results provided in Figure 10a also reveal that Figure 9 is the major structure after curing is complete. The cyano groups in BOZ-1N21 resin did not engage in the curing process and had no effect on the ring cleavage polymerization of the benzoxazine ring or the branching, crosslinking, and cyclization reactions of alkyne groups at the present temperature.

To compare the reaction rates of the above-mentioned two types of polymerization, the conversion rates (αt) of the functional groups at a specific temperature are based on the change in height of their characteristic peaks. The absorption peak (1761 cm^−1^) of the phthalide carbonyl group, which did not participate in any curing reaction, was chosen as the reference peak. *α_t_* was calculated by Equation (3), which could eliminate the effect of overlap peaks on the characteristic absorption peak.

(3) $$\alpha_{t}=\left[{\left(\frac{h_{x}}{h_{1761}}\right)}_{0}-{\left(\frac{h_{x}}{h_{1761}}\right)}_{t}\right]/\left[{\left(\frac{h_{x}}{h_{1761}}\right)}_{0}-{\left(\frac{h_{x}}{h_{1761}}\right)}_{f}\right]$$

Figure 10d depicts the conversion rate of the functional groups as a function of curing time at Tc = 180 °C, Tc = 208 °C, and Tc = 240 °C. The characteristic peaks of the alkyne groups −C−C− (2104 cm^−1^) and C−H (3280 cm^−1^) rise virtually concurrently, showing that the estimated result is correct. After curing the BOZ-1N21 resin at 180 °C for 2 h, the alkynyl−C−C and C−H values reached 0.43 and 0.42, respectively; after curing at 200 °C for 3 h, they rose by 0.36 and 0.37, respectively; and after curing at 240 °C for 3 h, they increased by 0.12 and 0.10. After curing at 180 °C for 2 h, the C−N−C value (1374 cm^−1^) associated with the benzoxazine ring reached 0.69, which is greater than that of the alkyne groups, indicating that the reactivity of the oxazine ring cleavage reaction is greater than that of the alkyne groups’ branched or trimerized cyclization reaction. In comparison to Xiong et al.’s [38] preliminary study, the cyano groups in BOZ-1N resin had no effect on the ring cleavage polymerization of benzoxazine or the cyclization process of alkynyl groups, corroborating the earlier findings.

### 3.5. Dynamic Mechanical Analysis of BOZ-1N Composite Material

**Figure 11:**
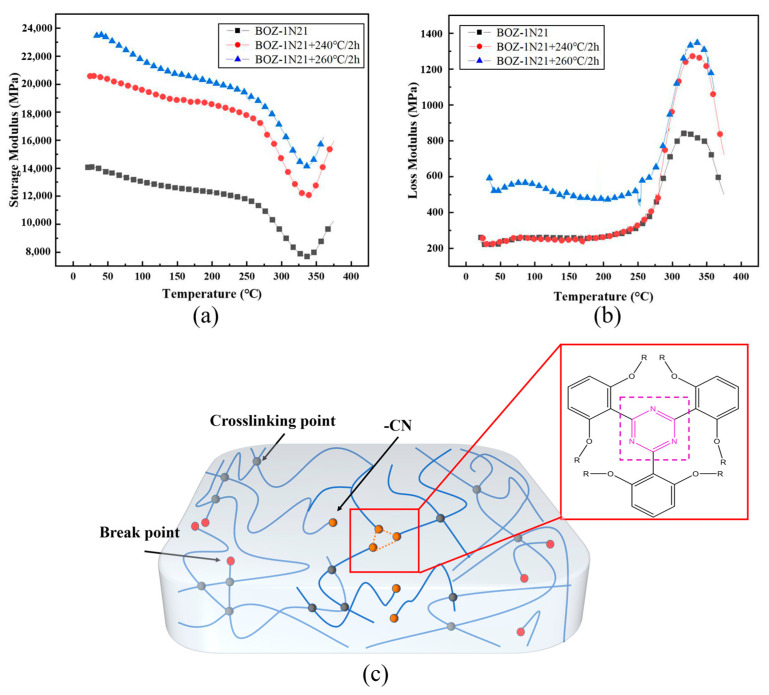
(**a**) Storage modulus-temperature curve of CF/BOZ-1N21 composite; (**b**) Loss modulus-temperature curve of CF/BOZ-1N21 composite; (**c**) Cyano groups curing crosslinking.

Based on the above-mentioned study of the curing temperature derived by DSC extrapolation, gelation time determined by isothermal DMA, and curing mechanism determined by in-situ isothermal FT-IR, the curing cycle is as follows: 180 °C/2 h, 208 °C/3 h, 240 °C/3 h, and two post-treatment procedures, 240 °C/2 h and 260 °C/2 h, were designed to explore the rationality of the curing process of CF/BOZ-1N composites, and the findings are given in Figure 11a. The storage modulus rises with post-curing time and temperature, as can be seen in Figure 11a. The storage modulus, for instance, can reach 24 GPa at room temperature following the 260 °C/2 h processing procedure. At 200 °C, the cured BOZ-1N composite material retained a high storage modulus of roughly 20 GPa. This means that there is some flexibility and mobility within the polymer network. However, this mobility does not result in a complete loss of mechanical stability. Continued heating to 275 °C resulted in an abrupt decrease in storage modulus; meanwhile, the loss modulus started to rise but did not peak as the temperature approached 275 °C, indicating a slight softening of the sample. It is worth noting that at a temperature of 330 °C, the storage modulus of the resin showed an increasing trend with the three curing processes rather than the usual downward trend. The storage modulus of BOZ-1N21 composite in this temperature range of 30 to 400 °C demonstrates that cured BOZ-1N21 has outstanding stiffness throughout a wide temperature range, which is advantageous for high temperature applications. The declining pattern of storage modulus of the three distinct curing procedures is congruent; as the temperature exceeds 250 °C, the rate of decline of storage modulus increases, most likely due to a decrease in the contact force between molecular chains as the temperature rises. At the same time, it demonstrates that the curing period has no major effect on the curing process or curing structure of the resin system.

When the curing temperature approaches 200 °C, a huge number of exothermic processes are thermally triggered, resulting in a substantially greater temperature in the system than 200 °C. When the reaction of the reactive groups is complete, the exterior temperature of the resin falls faster than the inside temperature, causing a large residual thermal stress in the system. The explanation for the rise in storage modulus after the 260 °C/2 h processing program is obvious: the high temperature reduces stress within the resin system, and the molecular chain arrangement is more ordered, resulting in an increase in energy storage modulus at the macro level. At temperatures over 325 °C, the cyano groups in the molecular structure form triazines with thermal stability, increasing the system’s crosslinking density and hence the energy storage modulus somewhat. Finally, a complete investigation indicated that the curing procedures of the CF/BOZ-1N composite were 180 °C/2 h, 208 °C/3 h, 240 °C/3 h, and 260 °C/2 h.

**Figure 12:**
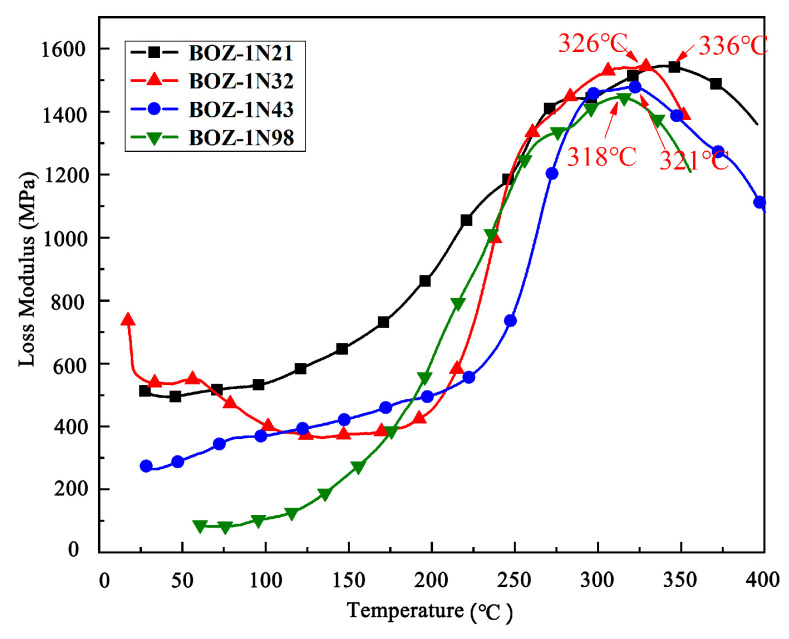
Loss modulus–temperature curves of four CF/BOZ-1N composites.

Figure 12 depicts the trend of loss modulus with increasing temperature for four different molecular weight CF/BOZ-1N composites cured at 180 °C/2 h, 208 °C/3 h, 240 °C/3 h, and 260 °C/2 h using the procedure described above. According to the result, the glass transition temperature (Tg) of the BOZ-1N resin decreases with increasing molecular weight. The higher the molecular weight, the more flexible the groups the BOZ-1N monomer contains and the lower the proportion of terminal alkyne groups, resulting in the growth of the flexible segment in the molecular chain, which decreases the crosslink density of the resin and thus leads to a decrease in Tg with molecular chain growth.

## 4. Conclusions

A novel alkynyl-functionalized benzoxazine with phthalide and cyano side groups has been created. The cyano group, as a latent crosslinking site, has no effect on the curing reaction of alkynyl and amphiphilic rings. When the temperature hits about 325 °C, the triazine ring generated by cyano crosslinking can improve the resin system’s energy storage modulus. The storage modulus of the monomer increases as its molecular weight grows, yet the toughness may theoretically be enhanced, and the influence on high-temperature resistance is less than 6%. These findings result in great thermal stability, a high Tg, and a high energy storage modulus for the produced resin, which is likely to be used in a high-temperature and impact-resistant composite material matrix.

## Figures and Tables

**Table 1:** Raw material ratios of four BOZ-1N resins with different molecular weights.

	PP:2,6-Dichlorobenzonitrile	PP (g)	2,6-Dichlorobenzonitrile (g)	K_2_CO_3_ (g)
1	2:1	63.6	17.2	27.6 g
2	3:2	95.4	34.4	41.4 g
3	4:3	63.6	25.8	27.6 g
4	9:8	57.24	27.52	24.84 g

**Table 2:** Curing characteristics of BOZ-1N21 resin at different heating rates.

Heating Rate (℃/min)	*T_i_* (°C)	*T_p_* (°C)	*T_f_* (°C)
5	184.11	214.18	248.46
10	197.47	224.38	262.71
15	201.05	232.66	268.59
20	202.16	236.75	280.06

Note: *T_i_* is the initial cure temperature, *T_p_* is the peak exothermic temperature, and *T_f_* is the final cure temperature.

## Data Availability

Supplemental data can be provided upon reasonable request.
